# (1R*,2S*)-*N*,*N*′-Bis[(*E*)-1*H*-pyrrol-2-yl­methyl­idene]cyclo­hexane-1,2-diamine monohydrate

**DOI:** 10.1107/S1600536812046193

**Published:** 2012-11-17

**Authors:** Kate J. Akerman

**Affiliations:** a School of Chemistry and Physics, University of KwaZulu-Natal, Private Bag X01, Scottsville, Pietermaritzburg, 3209, South Africa

## Abstract

The title compound, C_16_H_20_N_4_·H_2_O, was synthesized from *cis*-1,2-diamino­cyclo­hexane (a racemic mixture of the (1*R*,2*S*) and (1*S*,2*R*) enanti­omers). The compound crystallized with two mol­ecules (*A* and *B)* in the asymmetric unit with a single water solvent mol­ecule per Schiff base mol­ecule. Mol­ecules *A* and *B* have similar conformations as illustrated by the least-squares-fit with an r.m.s. deviation of 0.242 Å. The mol­ecules within the asymmetric unit are bridged by hydrogen bonds to the two water mol­ecules, resulting in a heterotetramer. The water mol­ecule acts as both a hydrogen-bond donor and acceptor. The pyrrole-imine units are not co-planar, making an angle of 73.9 (3)° and 76.9 (3)° in mol­ecules *A* and *B*, respectively.

## Related literature

For a study of the helical structures formed by both the *S*,*S* and *R*,*R* bis­(pyrrolide-imine) ligands as well as the Zn^II^, Cu^II^ and Ni^II^ chelates in the solid state, see: Wang *et al.* (2007 ▶). For the solid-state synthesis and X-ray structure of the anhydrous *trans* racemate of the ligand, see: van den Ancker *et al.* (2006 ▶). For the Ti^IV^ chelate of the *trans* racemic complex, see: Zhang *et al.* (2008 ▶). For the inter­molecular inter­action-controlled self-assembly and a study of the photophysics of the Pt^II^ chelate of the *R*,*R* and *S*,*S* enanti­omers as well as the *trans* racemic complex, see: Shan *et al.* (2008 ▶). For the X-ray structure and applications of the *trans* racemate of the Pd^II^ chelate as a hydrogenation catalyst, see: Bacchi *et al.* (2003 ▶). 
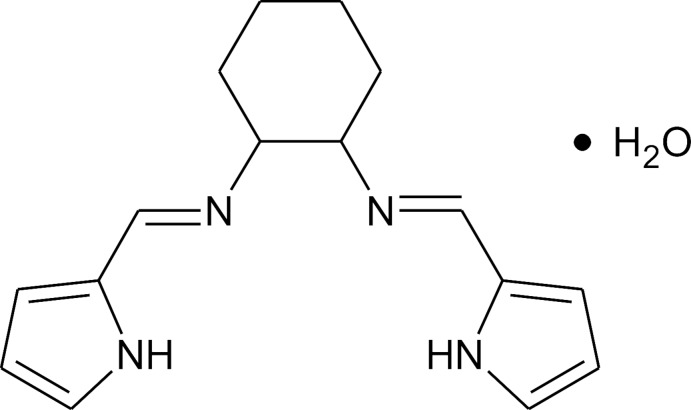


## Experimental

### 

#### Crystal data

C_16_H_20_N_4_·H_2_O
*M* *_r_* = 286.38
Monoclinic, 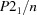

*a* = 9.7207 (7) Å
*b* = 18.4183 (13) Å
*c* = 18.2460 (12) Å
β = 92.721 (7)°
*V* = 3263.1 (4) Å^3^
*Z* = 8
Mo *K*α radiation
μ = 0.08 mm^−1^
*T* = 296 K
0.60 × 0.30 × 0.15 mm

#### Data collection

Oxford Diffraction Xcalibur 2 CCD diffractometer
Absorption correction: multi-scan (Blessing, 1995 ▶) *T*_min_ = 0.956, *T*_max_ = 0.989
23848 measured reflections
6428 independent reflections
3290 reflections with *I* > 2σ(*I*)
*R*_int_ = 0.067

#### Refinement

*R*[*F* ^2^ > 2σ(*F* ^2^)] = 0.050
*wR*(*F* ^2^) = 0.131
*S* = 0.85
6428 reflections
414 parameters
H atoms treated by a mixture of independent and constrained refinement
Δρ_max_ = 0.15 e Å^−3^
Δρ_min_ = −0.19 e Å^−3^

### 

Data collection: *CrysAlis CCD* (Oxford Diffraction, 2008 ▶); cell refinement: *CrysAlis CCD*; data reduction: *CrysAlis RED* (Oxford Diffraction, 2008 ▶); program(s) used to solve structure: *SHELXS97* (Sheldrick, 2008 ▶); program(s) used to refine structure: *SHELXL97* (Sheldrick, 2008 ▶); molecular graphics: *WinGX* (Farrugia, 2012 ▶); software used to prepare material for publication: *publCIF* (Westrip, 2010 ▶).

## Supplementary Material

Crystal structure: contains datablock(s) 1. DOI: 10.1107/S1600536812046193/vm2181sup1.cif

Additional supplementary materials:  crystallographic information; 3D view; checkCIF report

## Figures and Tables

**Table 1:** Hydrogen-bond geometry (Å, °)

*D*—H⋯*A*	*D*—H	H⋯*A*	*D*⋯*A*	*D*—H⋯*A*
O1*W*—H1*W*⋯N3*A*	0.82 (3)	2.28 (3)	3.014 (2)	149 (3)
O1*W*—H2*W*⋯N3*B*	0.92 (3)	1.96 (3)	2.857 (2)	166 (3)
O2*W*—H3*W*⋯N2*B*	0.80 (3)	2.16 (3)	2.927 (2)	159 (3)
O2*W*—H4*W*⋯N2*A*	0.98 (3)	1.88 (3)	2.819 (2)	159 (2)
N1*A*—H01*A*⋯O2*W*	0.93 (2)	2.03 (2)	2.896 (2)	154 (2)
N1*B*—H01*B*⋯O2*W*	0.95 (2)	1.96 (2)	2.899 (2)	169 (2)
N4*A*—H04*A*⋯O1*W*	0.88 (2)	2.02 (2)	2.882 (3)	166 (2)
N4*B*—H04*B*⋯O1*W*	0.86 (3)	2.09 (3)	2.896 (3)	155 (2)

**Fig. 1.**
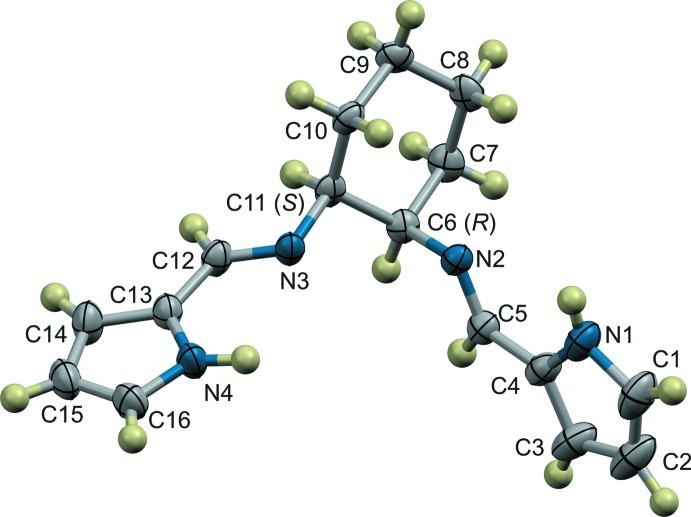
Thermal ellipsoid plot of molecule A of (1), rendered at 30% probability. Hydrogen atoms are shown as spheres of arbitrary radius. The solvent molecules and the second molecule of the asymmetric unit have been omitted for clarity.

**Fig. 2.**
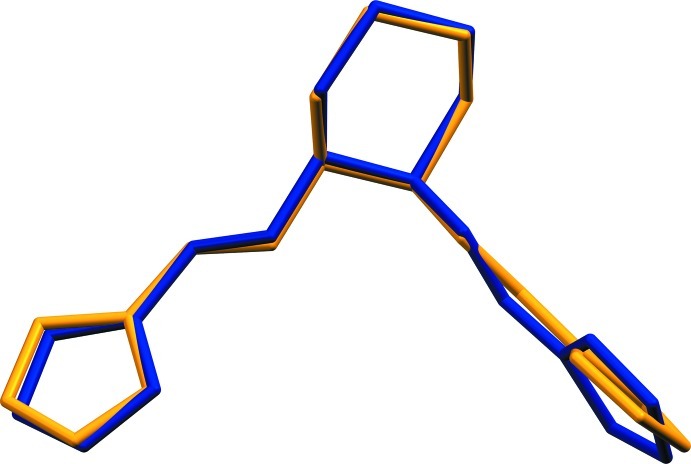
Least squares fit of molecules A and B, highlighting their similar geometries. Molecule A is shown in yellow and molecule B is shown in blue. RMSD = 0.242 Å.

**Fig. 3.**
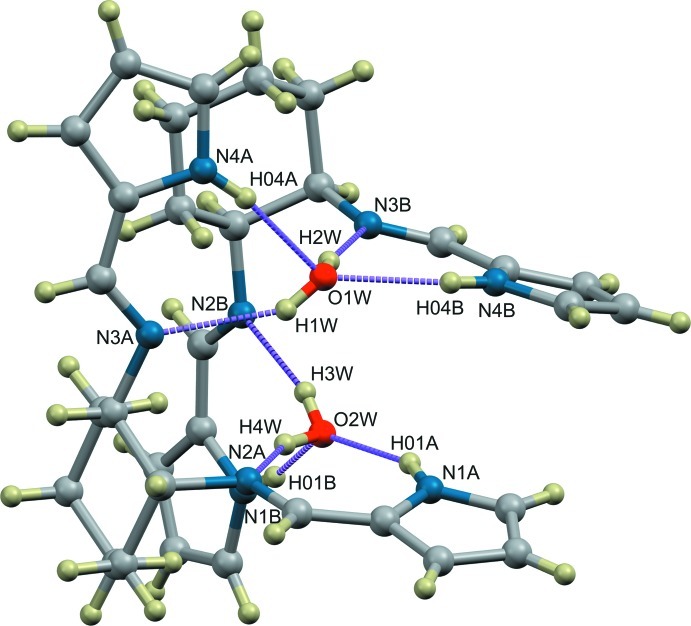
Contents of the asymmetric unit showing the water-bridged hydrogen-bonded dimer structure. Hydrogen bonds are shown as dashed, purple tubes. The atoms involved in hydrogen bonding have been labelled.

## supplementary crystallographic information

### Comment

The *R*,*R* and *S*,*S* enantiomers of the title compound have been extensively studied both as the metal chelate (Zn(II), Cu(II), Ni(II), Pd(II), Pt(II) and Ti(IV)) and the free ligand (Bacchi *et al.*, 2003; Shan *et al.*, 2008; van den Ancker *et al.*, 2006; Wang *et al.*, 2007 and Zhang *et al.*, 2008) while the mixed *R*,*S* enantiomer has received little attention. This is possibly owing to the fact that upon metal chelation the mean plane of the cyclohexyl ring is co-planar with the pyrrole-imine Schiff base moiety for the *R*,*R* and *S*,*S* enantiomers while in the case of the *R*,*S* enantiomer the mean plane of the cyclohexyl ring would be perpendicular to the pyrrole-imine Schiff base moiety; an unusual coordination geometry.

The asymmetric unit of the title compound comprises two molecules, A and B, and two water solvent molecules. The structure of molecule A showing the atom numbering scheme is shown in Figure 1. The geometry of molecules A and B are very similar, this is illustrated by a least squares fit (Figure 2) (Mercury, Macrae *et al.*, 2006). The RMSD for the fit is 0.242 Å. The fit shows that the biggest difference between the two structures is the torsion angle of the pyrrole rings relative to the cyclohexane linkage. The C6—N2—C4—N1 torsion angle is 179.7 (2) and 167.0 (2)° for molecules A and B, respectively. The C11—N3—C16—N4 torsion angle measures 173.5 (2) and 178.9 (2)° for molecules A and B, respectively. The mean imine C=N bond lengths are 1.270 (4) and 1.269 (3) Å for molecules A and B, respectively. These bond lengths highlight the double bond character of the imine bond. The pyrrole-imine moieties of both molecules A and B in the asymmetric unit are not co-planar. The angle subtended by the two seven atom mean planes comprising the pyrrole ring and imine carbon and nitrogen atoms is 73.9 (3)° and 76.9 (3)° for molecules A and B, respectively. This angle allows for hydrogen bonding to two water molecules. Both the imine nitrogen atoms and the pyrrole NH groups are involved in the hydrogen bonding, giving a total of eight hydrogen bonds. The hydrogen bonds result in a water-bridged dimer structure (Figure 3). The hydrogen bonds are considerably shorter than the sum of the van der Waals radii and the bond angles are approaching ideality, suggesting that they are likely to be relatively strong interactions. The hydrogen bond lengths and bond angles are summarized in Table 1.

### Experimental

The enatiomerically pure diamine, (1*R*,2*S*)-diaminocyclohexane, (0.303 g, 2.65 mmol) was ground in an agate pestle and mortar with pyrrole-2-carboxaldehyde (0.500 g, 5.30 mmol) for 10 minutes. The resulting brown oil was dissolved in dichloromethane and dried over magnesium sulfate to remove the water, a by-product from the condensation reaction. The dichloromethane solution was then concentrated and layered with hexane to re-crystallize the ligand by liquid-liquid difussion (0.512 g, 72% yield). Crystals suitable for single-crystal X-ray crystallography, were obtained from the crystallization process.

### Refinement

The positions of all C-bonded hydrogen atoms were calculated using the standard riding model of *SHELXL97* (Sheldrick, 2008) with C—H(aromatic) distances of 0.95 Å and *U*_iso_ = 1.2 *U*_eq_, *C*—*H*(methylene) distances of 0.99 Å and *U*_iso_ = 1.2 *U*_eq_ and a C—H(methine) distance of 1.00 Å and *U*_iso_ = 1.2 *U*_eq_. The pyrrole NH atoms and the hydrogen atoms of the water molecules were located in the difference density map and allowed to refine isotropically.

### Crystal data

**Table:**

C_16_H_20_N_4_·H_2_O	*F*(000) = 1232
*M**_r_* = 286.38	*D*_x_ = 1.166 Mg m^−^^3^
Monoclinic, *P*2_1_/*n*	Mo *K*α radiation, λ = 0.71073 Å
Hall symbol: -P 2yn	Cell parameters from 3290 reflections
*a* = 9.7207 (7) Å	θ = 3.0–26.0°
*b* = 18.4183 (13) Å	µ = 0.08 mm^−^^1^
*c* = 18.2460 (12) Å	*T* = 296 K
β = 92.721 (7)°	Needle, colourless
*V* = 3263.1 (4) Å^3^	0.60 × 0.30 × 0.15 mm
*Z* = 8	

### Data collection

**Table:**

Oxford Diffraction Xcalibur 2 CCD diffractometer	6428 independent reflections
Radiation source: fine-focus sealed tube	3290 reflections with *I* > 2σ(*I*)
Graphite monochromator	*R*_int_ = 0.067
ω scans at fixed θ angles	θ_max_ = 26.1°, θ_min_ = 3.1°
Absorption correction: multi-scan (Blessing, 1995)	*h* = −9→12
*T*_min_ = 0.956, *T*_max_ = 0.989	*k* = −22→22
23848 measured reflections	*l* = −22→22

### Refinement

**Table:**

Refinement on *F*^2^	Primary atom site location: structure-invariant direct methods
Least-squares matrix: full	Secondary atom site location: difference Fourier map
*R*[*F*^2^ > 2σ(*F*^2^)] = 0.050	Hydrogen site location: inferred from neighbouring sites
*wR*(*F*^2^) = 0.131	H atoms treated by a mixture of independent and constrained refinement
*S* = 0.85	*w* = 1/[σ^2^(*F*_o_^2^) + (0.0682*P*)^2^] where *P* = (*F*_o_^2^ + 2*F*_c_^2^)/3
6428 reflections	(Δ/σ)_max_ = 0.001
414 parameters	Δρ_max_ = 0.15 e Å^−^^3^
0 restraints	Δρ_min_ = −0.19 e Å^−^^3^

### Special details

**Table:**

Geometry. All e.s.d.'s (except the e.s.d. in the dihedral angle between two l.s. planes) are estimated using the full covariance matrix. The cell e.s.d.'s are taken into account individually in the estimation of e.s.d.'s in distances, angles and torsion angles; correlations between e.s.d.'s in cell parameters are only used when they are defined by crystal symmetry. An approximate (isotropic) treatment of cell e.s.d.'s is used for estimating e.s.d.'s involving l.s. planes.
Refinement. Refinement of *F*^2^ against ALL reflections. The weighted *R*-factor *wR* and goodness of fit *S* are based on *F*^2^, conventional *R*-factors *R* are based on *F*, with *F* set to zero for negative *F*^2^. The threshold expression of *F*^2^ > σ(*F*^2^) is used only for calculating *R*-factors(gt) *etc*. and is not relevant to the choice of reflections for refinement. *R*-factors based on *F*^2^ are statistically about twice as large as those based on *F*, and *R*- factors based on ALL data will be even larger.

### Fractional atomic coordinates and isotropic or equivalent isotropic displacement parameters (Å^2^)

**Table:**

	*x*	*y*	*z*	*U*_iso_*/*U*_eq_	
C1A	0.1913 (3)	−0.02963 (13)	0.54953 (14)	0.0834 (7)	
H1A	0.1123	−0.0581	0.5457	0.100*	
C1B	0.1477 (4)	−0.14986 (14)	0.40497 (14)	0.0960 (8)	
H1B	0.2269	−0.1680	0.4291	0.115*	
C2A	0.2955 (3)	−0.03661 (17)	0.60134 (15)	0.1018 (9)	
H2A	0.3006	−0.0704	0.6393	0.122*	
C2B	0.0173 (4)	−0.17302 (14)	0.41394 (14)	0.0997 (9)	
H2B	−0.0088	−0.2100	0.4451	0.120*	
C3A	0.3926 (3)	0.01569 (18)	0.58726 (14)	0.1030 (9)	
H3A	0.4750	0.0233	0.6141	0.124*	
C3B	−0.0705 (3)	−0.13179 (13)	0.36854 (12)	0.0845 (7)	
H3B	−0.1659	−0.1360	0.3640	0.101*	
C4A	0.3457 (2)	0.05500 (14)	0.52605 (12)	0.0730 (6)	
C4B	0.0090 (3)	−0.08341 (11)	0.33145 (11)	0.0615 (6)	
C5A	0.4118 (2)	0.11473 (14)	0.49148 (12)	0.0766 (7)	
H5A	0.4998	0.1266	0.5098	0.092*	
C5B	−0.0379 (2)	−0.03242 (10)	0.27595 (10)	0.0549 (5)	
H5B	−0.1323	−0.0307	0.2648	0.066*	
C6A	0.4517 (2)	0.21372 (13)	0.41631 (11)	0.0730 (7)	
H6A	0.546 (2)	0.2008 (3)	0.4273 (3)	0.088*	
C6B	−0.0344 (2)	0.05047 (10)	0.17992 (9)	0.0503 (5)	
H6B	−0.1358 (19)	0.04579 (13)	0.18469 (13)	0.060*	
C7A	0.4180 (3)	0.28228 (16)	0.45948 (14)	0.1059 (10)	
H7A1	0.4237	0.2712	0.5115	0.127*	
H7A2	0.4863	0.3192	0.4505	0.127*	
C7B	0.0038 (2)	0.01703 (11)	0.10669 (10)	0.0626 (6)	
H7B1	−0.0159	−0.0346	0.1073	0.075*	
H7B2	−0.0527	0.0387	0.0673	0.075*	
C8A	0.2756 (4)	0.31233 (15)	0.43906 (16)	0.1124 (11)	
H8A1	0.2608	0.3563	0.4669	0.135*	
H8A2	0.2064	0.2772	0.4518	0.135*	
C8B	0.1533 (2)	0.02822 (11)	0.09196 (11)	0.0633 (6)	
H8B1	0.1725	0.0072	0.0448	0.076*	
H8B2	0.2101	0.0034	0.1293	0.076*	
C9A	0.2600 (3)	0.32906 (13)	0.35746 (16)	0.0992 (9)	
H9A1	0.1664	0.3446	0.3452	0.119*	
H9A2	0.3216	0.3683	0.3456	0.119*	
C9B	0.1900 (2)	0.10788 (11)	0.09191 (10)	0.0636 (6)	
H9B1	0.1415	0.1316	0.0509	0.076*	
H9B2	0.2881	0.1132	0.0858	0.076*	
C10A	0.2932 (2)	0.26221 (11)	0.31272 (12)	0.0688 (6)	
H10C	0.2228	0.2257	0.3191	0.083*	
H10D	0.2913	0.2752	0.2612	0.083*	
C10B	0.1521 (2)	0.14446 (10)	0.16339 (10)	0.0546 (5)	
H10A	0.2116	0.1261	0.2034	0.065*	
H10B	0.1675	0.1963	0.1596	0.065*	
C11B	0.0035 (2)	0.13095 (10)	0.18041 (10)	0.0504 (5)	
H11B	−0.0552 (12)	0.1553 (5)	0.1421 (8)	0.060*	
C12A	0.5241 (2)	0.17287 (11)	0.23370 (11)	0.0575 (5)	
H12A	0.5608	0.2187	0.2257	0.069*	
C12B	−0.0978 (2)	0.22135 (11)	0.25033 (11)	0.0580 (5)	
H12B	−0.1286	0.2390	0.2047	0.070*	
C13A	0.6098 (2)	0.12312 (12)	0.11422 (11)	0.0651 (6)	
H13A	0.6493	0.1647	0.0954	0.078*	
C13B	−0.1349 (2)	0.26176 (10)	0.31397 (11)	0.0568 (5)	
C14A	0.6021 (2)	0.05552 (14)	0.08030 (13)	0.0747 (7)	
H14A	0.6359	0.0437	0.0350	0.090*	
C14B	−0.2075 (2)	0.32555 (12)	0.31707 (14)	0.0760 (7)	
H14B	−0.2446	0.3514	0.2770	0.091*	
C15A	0.5365 (2)	0.01026 (13)	0.12521 (14)	0.0750 (7)	
H15A	0.5163	−0.0383	0.1158	0.090*	
C15B	−0.2160 (2)	0.34479 (12)	0.39040 (14)	0.0760 (7)	
H15B	−0.2592	0.3857	0.4084	0.091*	
C16A	0.54886 (19)	0.11759 (11)	0.18053 (10)	0.0542 (5)	
C16B	−0.1498 (2)	0.29299 (13)	0.43048 (13)	0.0773 (7)	
H16B	−0.1395	0.2920	0.4814	0.093*	
C11A	0.4329 (2)	0.23015 (11)	0.33462 (11)	0.0641 (6)	
H11A	0.5029	0.2659	0.3225	0.077*	
N1A	0.2222 (2)	0.02607 (10)	0.50414 (10)	0.0681 (5)	
N1B	−0.1010 (2)	0.24262 (10)	0.38442 (10)	0.0657 (5)	
N2A	0.36308 (17)	0.15331 (10)	0.43840 (9)	0.0634 (5)	
N2B	−0.02667 (16)	0.16364 (8)	0.25133 (8)	0.0515 (4)	
N3A	0.45633 (16)	0.16439 (8)	0.29072 (8)	0.0564 (4)	
N3B	0.03694 (15)	0.01051 (8)	0.24065 (8)	0.0490 (4)	
N4A	0.50504 (19)	0.04740 (10)	0.18633 (10)	0.0622 (5)	
N4B	0.1423 (3)	−0.09489 (11)	0.35398 (10)	0.0752 (6)	
O1W	0.31430 (19)	0.01940 (10)	0.29915 (9)	0.0662 (4)	
O2W	0.08724 (17)	0.12068 (8)	0.39628 (9)	0.0597 (4)	
H01A	0.163 (2)	0.0437 (12)	0.4667 (13)	0.084 (7)*	
H01B	−0.045 (2)	0.2011 (13)	0.3941 (12)	0.088 (8)*	
H1W	0.344 (3)	0.0587 (18)	0.3136 (18)	0.143 (15)*	
H2W	0.227 (3)	0.0243 (14)	0.2780 (15)	0.111 (10)*	
H3W	0.067 (3)	0.1230 (13)	0.3531 (15)	0.097 (10)*	
H04A	0.452 (2)	0.0314 (10)	0.2203 (11)	0.060 (6)*	
H04B	0.213 (3)	−0.0690 (14)	0.3444 (14)	0.093 (9)*	
H4W	0.181 (3)	0.1399 (14)	0.4011 (14)	0.116 (10)*	

### Atomic displacement parameters (Å^2^)

**Table:**

	*U*^11^	*U*^22^	*U*^33^	*U*^12^	*U*^13^	*U*^23^
C1A	0.1007 (19)	0.0744 (16)	0.0764 (17)	0.0119 (14)	0.0177 (16)	0.0254 (14)
C1B	0.135 (3)	0.0733 (17)	0.0773 (18)	0.0007 (17)	−0.0223 (17)	0.0273 (14)
C2A	0.116 (2)	0.119 (2)	0.0715 (19)	0.0344 (19)	0.0133 (18)	0.0394 (16)
C2B	0.159 (3)	0.0757 (18)	0.0619 (17)	−0.0357 (19)	−0.0142 (18)	0.0187 (13)
C3A	0.0846 (18)	0.164 (3)	0.0595 (16)	0.0235 (19)	−0.0083 (14)	0.0293 (17)
C3B	0.115 (2)	0.0806 (17)	0.0571 (14)	−0.0313 (15)	−0.0029 (14)	0.0140 (13)
C4A	0.0652 (14)	0.1068 (19)	0.0467 (13)	0.0077 (14)	−0.0012 (11)	0.0139 (13)
C4B	0.0913 (17)	0.0508 (13)	0.0424 (12)	−0.0126 (11)	0.0028 (12)	−0.0006 (10)
C5A	0.0606 (13)	0.123 (2)	0.0460 (13)	−0.0104 (14)	−0.0041 (11)	−0.0040 (14)
C5B	0.0678 (13)	0.0512 (12)	0.0453 (11)	−0.0057 (10)	−0.0019 (10)	−0.0054 (10)
C6A	0.0754 (14)	0.0983 (18)	0.0458 (12)	−0.0344 (13)	0.0070 (11)	−0.0200 (12)
C6B	0.0536 (11)	0.0563 (13)	0.0399 (11)	0.0028 (9)	−0.0095 (9)	−0.0005 (9)
C7A	0.150 (3)	0.115 (2)	0.0548 (15)	−0.068 (2)	0.0248 (17)	−0.0360 (15)
C7B	0.0786 (15)	0.0648 (14)	0.0428 (12)	−0.0001 (11)	−0.0125 (10)	−0.0110 (10)
C8A	0.161 (3)	0.0830 (19)	0.098 (2)	−0.038 (2)	0.059 (2)	−0.0525 (16)
C8B	0.0810 (15)	0.0716 (15)	0.0368 (11)	0.0136 (11)	−0.0026 (10)	−0.0119 (10)
C9A	0.139 (2)	0.0598 (15)	0.102 (2)	−0.0112 (15)	0.0414 (19)	−0.0284 (14)
C9B	0.0746 (14)	0.0794 (16)	0.0368 (11)	0.0086 (11)	0.0044 (10)	0.0102 (10)
C10A	0.0991 (18)	0.0484 (12)	0.0598 (14)	−0.0084 (12)	0.0138 (12)	−0.0085 (10)
C10B	0.0754 (13)	0.0478 (11)	0.0401 (11)	−0.0042 (10)	−0.0012 (10)	0.0064 (9)
C11B	0.0691 (13)	0.0499 (12)	0.0312 (10)	0.0088 (10)	−0.0072 (9)	0.0047 (8)
C12A	0.0641 (12)	0.0622 (13)	0.0465 (12)	−0.0104 (10)	0.0053 (10)	−0.0036 (10)
C12B	0.0776 (14)	0.0500 (12)	0.0454 (12)	0.0067 (11)	−0.0061 (10)	0.0032 (9)
C13A	0.0718 (14)	0.0757 (15)	0.0491 (12)	−0.0018 (11)	0.0163 (11)	−0.0016 (11)
C13B	0.0666 (13)	0.0474 (12)	0.0562 (13)	0.0080 (10)	0.0004 (10)	−0.0043 (10)
C14A	0.0742 (15)	0.0912 (18)	0.0603 (15)	0.0071 (13)	0.0209 (12)	−0.0173 (13)
C14B	0.0962 (17)	0.0586 (14)	0.0720 (16)	0.0218 (12)	−0.0082 (13)	0.0002 (12)
C15A	0.0776 (15)	0.0657 (15)	0.0831 (17)	0.0064 (12)	0.0172 (13)	−0.0204 (13)
C15B	0.0831 (16)	0.0575 (14)	0.0882 (19)	0.0126 (12)	0.0123 (14)	−0.0159 (13)
C16A	0.0593 (12)	0.0586 (13)	0.0449 (12)	−0.0013 (10)	0.0046 (10)	−0.0016 (10)
C16B	0.0989 (17)	0.0711 (16)	0.0631 (15)	0.0170 (14)	0.0171 (13)	−0.0071 (13)
C11A	0.0831 (15)	0.0620 (13)	0.0484 (12)	−0.0293 (12)	0.0154 (11)	−0.0167 (10)
N1A	0.0729 (13)	0.0778 (13)	0.0533 (11)	0.0068 (11)	0.0006 (10)	0.0151 (10)
N1B	0.0864 (13)	0.0570 (11)	0.0545 (12)	0.0201 (10)	0.0112 (10)	−0.0006 (9)
N2A	0.0669 (11)	0.0879 (13)	0.0353 (9)	−0.0162 (9)	0.0004 (8)	−0.0047 (9)
N2B	0.0695 (10)	0.0470 (9)	0.0377 (9)	0.0084 (8)	−0.0005 (8)	0.0002 (7)
N3A	0.0692 (11)	0.0614 (11)	0.0394 (9)	−0.0114 (8)	0.0113 (8)	−0.0074 (8)
N3B	0.0602 (10)	0.0465 (9)	0.0399 (9)	−0.0024 (8)	−0.0024 (8)	−0.0005 (7)
N4A	0.0702 (11)	0.0622 (12)	0.0555 (11)	0.0013 (9)	0.0150 (10)	−0.0001 (9)
N4B	0.0966 (17)	0.0629 (13)	0.0653 (13)	−0.0041 (12)	−0.0064 (12)	0.0177 (10)
O1W	0.0643 (10)	0.0714 (12)	0.0625 (10)	−0.0013 (9)	−0.0032 (8)	0.0150 (8)
O2W	0.0682 (10)	0.0679 (10)	0.0427 (9)	0.0000 (7)	−0.0019 (8)	0.0094 (7)

### Geometric parameters (Å, º)

**Table:**

C1A—C2A	1.358 (3)	C9A—H9A2	0.9700
C1A—N1A	1.361 (3)	C9B—C10B	1.528 (3)
C1A—H1A	0.9300	C9B—H9B1	0.9700
C1B—C2B	1.355 (4)	C9B—H9B2	0.9700
C1B—N4B	1.374 (3)	C10A—C11A	1.517 (3)
C1B—H1B	0.9300	C10A—H10C	0.9700
C2A—C3A	1.381 (4)	C10A—H10D	0.9700
C2A—H2A	0.9300	C10B—C11B	1.512 (3)
C2B—C3B	1.387 (3)	C10B—H10A	0.9700
C2B—H2B	0.9300	C10B—H10B	0.9700
C3A—C4A	1.390 (3)	C11B—N2B	1.469 (2)
C3A—H3A	0.9300	C11B—H11B	0.9894
C3B—C4B	1.378 (3)	C12A—N3A	1.267 (2)
C3B—H3B	0.9300	C12A—C16A	1.435 (3)
C4A—N1A	1.356 (3)	C12A—H12A	0.9300
C4A—C5A	1.435 (3)	C12B—N2B	1.268 (2)
C4B—N4B	1.357 (3)	C12B—C13B	1.439 (3)
C4B—C5B	1.439 (3)	C12B—H12B	0.9300
C5A—N2A	1.274 (3)	C13A—C16A	1.376 (3)
C5A—H5A	0.9300	C13A—C14A	1.391 (3)
C5B—N3B	1.271 (2)	C13A—H13A	0.9300
C5B—H5B	0.9300	C13B—N1B	1.358 (2)
C6A—N2A	1.475 (3)	C13B—C14B	1.373 (3)
C6A—C11A	1.523 (3)	C14A—C15A	1.350 (3)
C6A—C7A	1.532 (3)	C14A—H14A	0.9300
C6A—H6A	0.9609	C14B—C15B	1.390 (3)
C6B—N3B	1.476 (2)	C14B—H14B	0.9300
C6B—C11B	1.527 (3)	C15A—N4A	1.356 (3)
C6B—C7B	1.533 (3)	C15A—H15A	0.9300
C6B—H6B	0.9975	C15B—C16B	1.347 (3)
C7A—C8A	1.520 (4)	C15B—H15B	0.9300
C7A—H7A1	0.9700	C16A—N4A	1.367 (3)
C7A—H7A2	0.9700	C16B—N1B	1.353 (3)
C7B—C8B	1.505 (3)	C16B—H16B	0.9300
C7B—H7B1	0.9700	C11A—N3A	1.476 (2)
C7B—H7B2	0.9700	C11A—H11A	0.9800
C8A—C9A	1.521 (4)	N1A—H01A	0.93 (2)
C8A—H8A1	0.9700	N1B—H01B	0.95 (2)
C8A—H8A2	0.9700	N4A—H04A	0.88 (2)
C8B—C9B	1.510 (3)	N4B—H04B	0.86 (2)
C8B—H8B1	0.9700	O1W—H1W	0.82 (3)
C8B—H8B2	0.9700	O1W—H2W	0.92 (3)
C9A—C10A	1.520 (3)	O2W—H3W	0.81 (3)
C9A—H9A1	0.9700	O2W—H4W	0.98 (3)
			
C2A—C1A—N1A	108.4 (3)	C8B—C9B—H9B2	109.4
C2A—C1A—H1A	125.8	C10B—C9B—H9B2	109.4
N1A—C1A—H1A	125.8	H9B1—C9B—H9B2	108.0
C2B—C1B—N4B	107.9 (3)	C11A—C10A—C9A	112.6 (2)
C2B—C1B—H1B	126.0	C11A—C10A—H10C	109.1
N4B—C1B—H1B	126.0	C9A—C10A—H10C	109.1
C1A—C2A—C3A	107.3 (2)	C11A—C10A—H10D	109.1
C1A—C2A—H2A	126.3	C9A—C10A—H10D	109.1
C3A—C2A—H2A	126.3	H10C—C10A—H10D	107.8
C1B—C2B—C3B	107.9 (2)	C11B—C10B—C9B	111.97 (16)
C1B—C2B—H2B	126.0	C11B—C10B—H10A	109.2
C3B—C2B—H2B	126.0	C9B—C10B—H10A	109.2
C2A—C3A—C4A	108.2 (2)	C11B—C10B—H10B	109.2
C2A—C3A—H3A	125.9	C9B—C10B—H10B	109.2
C4A—C3A—H3A	125.9	H10A—C10B—H10B	107.9
C4B—C3B—C2B	107.7 (2)	N2B—C11B—C10B	110.24 (15)
C4B—C3B—H3B	126.2	N2B—C11B—C6B	110.17 (14)
C2B—C3B—H3B	126.2	C10B—C11B—C6B	113.02 (15)
N1A—C4A—C3A	106.5 (2)	N2B—C11B—H11B	107.7
N1A—C4A—C5A	125.5 (2)	C10B—C11B—H11B	107.7
C3A—C4A—C5A	128.0 (3)	C6B—C11B—H11B	107.7
N4B—C4B—C3B	107.5 (2)	N3A—C12A—C16A	125.31 (19)
N4B—C4B—C5B	125.49 (19)	N3A—C12A—H12A	117.3
C3B—C4B—C5B	127.0 (2)	C16A—C12A—H12A	117.3
N2A—C5A—C4A	127.1 (2)	N2B—C12B—C13B	125.39 (18)
N2A—C5A—H5A	116.4	N2B—C12B—H12B	117.3
C4A—C5A—H5A	116.4	C13B—C12B—H12B	117.3
N3B—C5B—C4B	126.36 (19)	C16A—C13A—C14A	108.0 (2)
N3B—C5B—H5B	116.8	C16A—C13A—H13A	126.0
C4B—C5B—H5B	116.8	C14A—C13A—H13A	126.0
N2A—C6A—C11A	111.87 (16)	N1B—C13B—C14B	106.57 (18)
N2A—C6A—C7A	109.82 (18)	N1B—C13B—C12B	124.79 (18)
C11A—C6A—C7A	108.7 (2)	C14B—C13B—C12B	128.6 (2)
N2A—C6A—H6A	108.8	C15A—C14A—C13A	107.43 (19)
C11A—C6A—H6A	108.8	C15A—C14A—H14A	126.3
C7A—C6A—H6A	108.8	C13A—C14A—H14A	126.3
N3B—C6B—C11B	112.01 (14)	C13B—C14B—C15B	108.2 (2)
N3B—C6B—C7B	109.23 (15)	C13B—C14B—H14B	125.9
C11B—C6B—C7B	109.08 (15)	C15B—C14B—H14B	125.9
N3B—C6B—H6B	108.8	C14A—C15A—N4A	108.6 (2)
C11B—C6B—H6B	108.8	C14A—C15A—H15A	125.7
C7B—C6B—H6B	108.8	N4A—C15A—H15A	125.7
C8A—C7A—C6A	112.9 (2)	C16B—C15B—C14B	107.0 (2)
C8A—C7A—H7A1	109.0	C16B—C15B—H15B	126.5
C6A—C7A—H7A1	109.0	C14B—C15B—H15B	126.5
C8A—C7A—H7A2	109.0	N4A—C16A—C13A	106.68 (18)
C6A—C7A—H7A2	109.0	N4A—C16A—C12A	123.78 (18)
H7A1—C7A—H7A2	107.8	C13A—C16A—C12A	129.4 (2)
C8B—C7B—C6B	112.12 (16)	C15B—C16B—N1B	108.7 (2)
C8B—C7B—H7B1	109.2	C15B—C16B—H16B	125.6
C6B—C7B—H7B1	109.2	N1B—C16B—H16B	125.6
C8B—C7B—H7B2	109.2	N3A—C11A—C10A	109.62 (16)
C6B—C7B—H7B2	109.2	N3A—C11A—C6A	110.71 (18)
H7B1—C7B—H7B2	107.9	C10A—C11A—C6A	113.55 (18)
C7A—C8A—C9A	111.2 (2)	N3A—C11A—H11A	107.6
C7A—C8A—H8A1	109.4	C10A—C11A—H11A	107.6
C9A—C8A—H8A1	109.4	C6A—C11A—H11A	107.6
C7A—C8A—H8A2	109.4	C4A—N1A—C1A	109.6 (2)
C9A—C8A—H8A2	109.4	C4A—N1A—H01A	126.1 (14)
H8A1—C8A—H8A2	108.0	C1A—N1A—H01A	124.1 (14)
C7B—C8B—C9B	111.30 (17)	C16B—N1B—C13B	109.48 (19)
C7B—C8B—H8B1	109.4	C16B—N1B—H01B	130.6 (14)
C9B—C8B—H8B1	109.4	C13B—N1B—H01B	119.7 (14)
C7B—C8B—H8B2	109.4	C5A—N2A—C6A	115.31 (19)
C9B—C8B—H8B2	109.4	C12B—N2B—C11B	117.55 (15)
H8B1—C8B—H8B2	108.0	C12A—N3A—C11A	116.37 (16)
C10A—C9A—C8A	110.3 (2)	C5B—N3B—C6B	115.52 (16)
C10A—C9A—H9A1	109.6	C15A—N4A—C16A	109.28 (19)
C8A—C9A—H9A1	109.6	C15A—N4A—H04A	125.2 (13)
C10A—C9A—H9A2	109.6	C16A—N4A—H04A	124.7 (13)
C8A—C9A—H9A2	109.6	C4B—N4B—C1B	109.0 (2)
H9A1—C9A—H9A2	108.1	C4B—N4B—H04B	127.7 (17)
C8B—C9B—C10B	111.18 (15)	C1B—N4B—H04B	122.7 (18)
C8B—C9B—H9B1	109.4	H1W—O1W—H2W	110 (3)
C10B—C9B—H9B1	109.4	H3W—O2W—H4W	105 (2)
			
N1A—C1A—C2A—C3A	−0.2 (3)	C14A—C13A—C16A—N4A	0.0 (2)
N4B—C1B—C2B—C3B	−0.4 (3)	C14A—C13A—C16A—C12A	−176.2 (2)
C1A—C2A—C3A—C4A	0.2 (3)	N3A—C12A—C16A—N4A	−2.1 (3)
C1B—C2B—C3B—C4B	0.3 (3)	N3A—C12A—C16A—C13A	173.5 (2)
C2A—C3A—C4A—N1A	−0.1 (3)	C14B—C15B—C16B—N1B	0.0 (3)
C2A—C3A—C4A—C5A	179.8 (2)	C9A—C10A—C11A—N3A	178.26 (18)
C2B—C3B—C4B—N4B	−0.1 (3)	C9A—C10A—C11A—C6A	53.9 (2)
C2B—C3B—C4B—C5B	176.8 (2)	N2A—C6A—C11A—N3A	−55.2 (2)
N1A—C4A—C5A—N2A	5.0 (4)	C7A—C6A—C11A—N3A	−176.66 (18)
C3A—C4A—C5A—N2A	−174.8 (3)	N2A—C6A—C11A—C10A	68.6 (2)
N4B—C4B—C5B—N3B	−1.5 (3)	C7A—C6A—C11A—C10A	−52.9 (2)
C3B—C4B—C5B—N3B	−177.9 (2)	C3A—C4A—N1A—C1A	0.0 (3)
N2A—C6A—C7A—C8A	−67.8 (3)	C5A—C4A—N1A—C1A	−179.9 (2)
C11A—C6A—C7A—C8A	54.9 (3)	C2A—C1A—N1A—C4A	0.2 (3)
N3B—C6B—C7B—C8B	−66.8 (2)	C15B—C16B—N1B—C13B	0.3 (3)
C11B—C6B—C7B—C8B	56.0 (2)	C14B—C13B—N1B—C16B	−0.4 (2)
C6A—C7A—C8A—C9A	−57.4 (3)	C12B—C13B—N1B—C16B	179.0 (2)
C6B—C7B—C8B—C9B	−57.8 (2)	C4A—C5A—N2A—C6A	177.1 (2)
C7A—C8A—C9A—C10A	54.9 (3)	C11A—C6A—N2A—C5A	148.9 (2)
C7B—C8B—C9B—C10B	55.1 (2)	C7A—C6A—N2A—C5A	−90.3 (3)
C8A—C9A—C10A—C11A	−53.4 (3)	C13B—C12B—N2B—C11B	−178.54 (18)
C8B—C9B—C10B—C11B	−52.8 (2)	C10B—C11B—N2B—C12B	105.68 (19)
C9B—C10B—C11B—N2B	176.98 (15)	C6B—C11B—N2B—C12B	−128.92 (18)
C9B—C10B—C11B—C6B	53.2 (2)	C16A—C12A—N3A—C11A	−174.75 (18)
N3B—C6B—C11B—N2B	−56.5 (2)	C10A—C11A—N3A—C12A	95.5 (2)
C7B—C6B—C11B—N2B	−177.59 (15)	C6A—C11A—N3A—C12A	−138.48 (19)
N3B—C6B—C11B—C10B	67.27 (19)	C4B—C5B—N3B—C6B	172.32 (17)
C7B—C6B—C11B—C10B	−53.8 (2)	C11B—C6B—N3B—C5B	134.21 (17)
N2B—C12B—C13B—N1B	−1.5 (3)	C7B—C6B—N3B—C5B	−104.84 (19)
N2B—C12B—C13B—C14B	177.8 (2)	C14A—C15A—N4A—C16A	0.9 (3)
C16A—C13A—C14A—C15A	0.5 (3)	C13A—C16A—N4A—C15A	−0.5 (2)
N1B—C13B—C14B—C15B	0.4 (3)	C12A—C16A—N4A—C15A	175.93 (18)
C12B—C13B—C14B—C15B	−179.0 (2)	C3B—C4B—N4B—C1B	−0.1 (3)
C13A—C14A—C15A—N4A	−0.8 (3)	C5B—C4B—N4B—C1B	−177.1 (2)
C13B—C14B—C15B—C16B	−0.3 (3)	C2B—C1B—N4B—C4B	0.4 (3)

### Hydrogen-bond geometry (Å, º)

**Table:**

*D*—H···*A*	*D*—H	H···*A*	*D*···*A*	*D*—H···*A*
O1*W*—H1*W*···N3*A*	0.82 (3)	2.28 (3)	3.014 (2)	149 (3)
O1*W*—H2*W*···N3*B*	0.92 (3)	1.96 (3)	2.857 (2)	166 (3)
O2*W*—H3*W*···N2*B*	0.80 (3)	2.16 (3)	2.927 (2)	159 (3)
O2*W*—H4*W*···N2*A*	0.98 (3)	1.88 (3)	2.819 (2)	159 (2)
N1*A*—H01*A*···O2*W*	0.93 (2)	2.03 (2)	2.896 (2)	154 (2)
N1*B*—H01*B*···O2*W*	0.95 (2)	1.96 (2)	2.899 (2)	169 (2)
N4*A*—H04*A*···O1*W*	0.88 (2)	2.02 (2)	2.882 (3)	166 (2)
N4*B*—H04*B*···O1*W*	0.86 (3)	2.09 (3)	2.896 (3)	155 (2)
